# 3,3′-Thiodipropionic acid (TDP), a possible precursor for the synthesis of polythioesters: identification of TDP transport proteins in *Variovorax paradoxus* TBEA6

**DOI:** 10.1007/s00253-021-11294-y

**Published:** 2021-04-26

**Authors:** M. Venkateswar Reddy, Alexander Steinbüchel

**Affiliations:** 1 Institut für Molekulare Mikrobiologie und Biotechnologie, Westfälische Wilhelms-Universität Münster, 48149 Münster, Germany; 2 Environmental Sciences Department, King Abdulaziz University, Jeddah, Saudi Arabia

**Keywords:** Deletion mutant, Polythioesters, Protein purification, Thermoshift assay, 3,3′-Thiodipropionic acid (TDP), *Variovorax paradoxus*

## Abstract

3,3′-Thiodipropionic acid (TDP) is an antioxidant, which can be used as precursor carbon source to synthesize polythioesters. The bacterium *Variovorax paradoxus* TBEA6 strain can use TDP as a single source of carbon and energy. In the present study, experiments were carried out to identify proteins involved in the transport of TDP into the cells of strain TBEA6. Hence, eight putative *tctC* genes, which encode for the TctC proteins, were amplified from genomic DNA of TBEA6 strain using polymerase chain reaction and expressed in *E. coli* BL21 cells. Cells were grown in auto-induction medium, and protein purification was done using His Spin Trap affinity columns. Purity and molecular weight of each protein were confirmed by SDS-PAGE analysis. Protein-ligand interactions were monitored in thermoshift assays using the real-time PCR system. Two TctC proteins (locus tags VPARA-44430 and VPARA-01760) out of eight proteins showed a significant shift in their melting temperatures when they interact with the ligand (TDP or gluconate). The responsible genes were deleted in the genome of TBEA6 using suicide plasmid pJQ200mp18Tc, and single deletion mutants of the two candidate genes were subsequently generated. Finally, growth of the wild-type strain (TBEA6) and the two mutant strains (ΔVPARA-44430 and ΔVPARA-01760) were monitored and compared using TDP or gluconate as carbon sources. Wild type strains were successfully grown with TDP or gluconate. From the two mutant strains, one (ΔVPARA-44430) was unable to grow with TDP indicating that the *tctC* gene with locus tag VPARA-44430 is involved in the uptake of TDP.

Key Points

*• Putative tctC genes from V. paradoxus TBEA6 were heterologously expressed in E. coli.*

*• Protein-ligand interactions monitored in thermoshift assays using the real-time PCR.*

*• tctC gene with locus tag VPARA-44430 is involved in the uptake of TDP.*

**Supplementary Information:**

The online version contains supplementary material available at 10.1007/s00253-021-11294-y.

## Introduction

Members of Gram-negative, aerobic betaproteobacteria belonging to the genus *Variovorax* are exist in soil and water (Rodriguez et al. 2011). *Alcaligenes* was renamed as genus *Variovorax*, and the cells can accumulate poly(3-hydroxybutyric acid) (Satola et al. 2013). Due to its various metabolic competences, *Variovorax paradoxus* has auspicious selections for applications in bioremediation because it is able to degrade organic sulfur compounds, aromatic compounds, polymers, 2,4-dichlorophenoxyacetic acid, 2,4-dinitrotoluene, and resists metal ions (Satola et al. 2013; Schürmann et al. 2013). In addition, this bacterium can grow on simple carbohydrates like glucose, mannose, and galactose (Schürmann et al. 2013). Various strains of *V. paradoxus* were already isolated and reported for their suitability to be used in different applications.

*V. paradoxus* strain TBEA6 was isolated and evaluated for its 3,3′-thiodipropionic acid (TDP) degradation capability (Bruland et al. 2009). TDP is an important additive of polyolefins (Satola et al. 2013) and was used as a carbon source to produce polythioesters (PTE) in the bacterium *Ralstonia eutropha*. Sulfur containing PTE homopolymers are biologically synthesized and persistent, non-biodegradable polymers (Lütke-Eversloh and Steinbüchel 2004; Kim et al. 2005). Previously, PTE production was restricted due to its dependence on expensive and toxic raw materials like 3-mercaptopropionic acid (Lütke-Eversloh and Steinbüchel 2004; Xia et al. 2012). Hence, research related to PTE production using TDP as raw material and the pathways involved in TDP degradation was initiated (Bruland et al. 2009). Accordingly, the genome of TBEA6 was sequenced, and transposon mutagenesis to explore the catabolism of TDP was done (Wübbeler et al. 2015). The information gained through annotated genome sequence together with the analyses of transposon-induced mutants, specific gene deletions, and in silico analysis indicated that TDP is transported through the tripartite tricarboxylate transport (TTT) or the TRAP transport system.

The TTT system initially explored in *Salmonella typhimurium* consists of a three component binding protein-based (TctA, TctB, and TctC) transport systems, associated with the two regulatory components TctD and TctE (Winnen et al. 2003; Huvent et al. 2006; Schäfer et al. 2019). TctA and TctB are membrane proteins but possess different conserved motifs (Winnen et al. 2003; Tamber et al. 2006). TctC is a periplasmic-binding receptor protein which binds to the substrate and facilitates the import supported by TctA and TctB (Huvent et al. 2006). The transport is generally regulated by TctD and TctE. Nicotinic acid, negatively charged amino acids, isocitrate, and fluoro citrate were imported by TTT systems (Antoine et al. 2003, 2005).

Hosaka et al. (2013) reported that isophthalic acid, terephthalic acid, and trimesic acid were transported by these exciting ATP independent transport systems. Rosa et al. (2017, 2019) reported that adipate and malate are substrates for the proteins AdpC and MatC, respectively, of a TTT system present in *Rhodopseudomonas palustris*. TTT systems are also involved in the transport of citrate in *Advenella mimigardefordensis* DPN7T (Schäfer et al. 2019). The aim of this study is to identify the proteins involved in the transport of TDP. Hence, out of 130 homologues, eight TctC proteins, which exhibit more than 40% similarity to AdpC proteins, were selected; the responsible genes were amplified from genomic DNA of TBEA6 and heterologously expressed in *E. coli* BL21 cells. After protein purification, protein-ligand interactions were monitored in thermoshift assays using real-time PCR. The responsible genes were deleted in the genome of TBEA6, and growth of the wild-type strain and the mutant strains (ΔVPARA-44430 and ΔVPARA-01760) were monitored using TDP or gluconate.

## Materials and methods

### Bacterial cultures

*V. paradoxus* TBEA6 was previously isolated from soil (Bruland et al. 2009). Cells of *V. paradoxus* TBEA6 were cultivated at 30 °C on solid MSM agar plates (Schlegel et al. 1961) containing gluconate or TDP to test growth on the carbon sources. Table 1 shows the list of strains used in this study. *E. coli* cells were grown with lysogeny broth (LB) medium at 37 °C (Sambrook et al. 1989). *E. coli* Top10 and BL21 cells were used for cloning and protein expression, respectively. Carbon sources were supplied as filter sterilized 1 M stock solutions after adjusting the pH to 7. Purified agar (2%) was used for the preparation of solid media. Highly pure TDP was procured from Sigma-Aldrich (Steinheim, Germany). For the maintenance of plasmids, antibiotics (ampicillin-75 μg/ml and tetracycline-12.5 μg/ml) were added (Sambrook et al. 1989). *E. coli* S17-1 was used for the transfer of DNA to *V. paradoxus* TBEA6 via conjugation. Bruland et al. (2009) published the first report dealing with *V. paradoxus* TBEA6 and the primary results about genes involved in TDP catabolism. Nucleotide sequence of the 16 S rRNA gene of *V. paradoxus* TBEA6 was deposited in the GenBank (accession number: EF641108) (Bruland et al. 2009).

**Table 1:** Strains and plasmids used in this study

Name of strain/or plasmid	Description of relevant phenotype or genotype	Reference
**Strains**		
*V. paradoxus* TBEA6	Wild type — uses TDP and citrate as the sole carbon source	Bruland et al. 2009
*V. paradoxus* TBEA6ΔtctC-44430	Precise deletion mutant of *V. paradoxus* TBEA6, lacs VPARA_44430	This study
*V. paradoxus* TBEA6ΔtctC-01760	Precise deletion mutant of *V. paradoxus* TBEA6, lacs VPARA_01760	This study
*E. coli* TOP10	F^−^, *mcrA*, Δ (*mrr*-*hsd*RMS-*mcr*BC), *rps*L, *nupG*, Φ80lacZΔM15, Δ*lac*X74, *deo*R, *rec*A1, *ara*D139, Δ(*ara*-*leu*)7697, galU, *gal*K, *end*A1	Invitrogen, Germany
*E. coli* BL21 (DE3) (pLysS)	F^−^*ompThsdS*_B_ (rB^−^ mB^−^) *gal dcm* (DE3) /pLysS (Cmr)	Novagen, USA
S17-1	*thi*-1, *proA*, *hsd* R17 (rk^−^ mk^+^), *rec*A1, *tra*-genes of plasmid RP4 integrated into the genome F^−^, *mcr*A, Δ(*mrr-hsd*RMS-*mcr*BC), *rps*L, *nup*G	Simon et al. 1983
Plasmids		
pET19b	*E. coli* expression vector, (N-terminal His-tag, Amp^r^, T7 promoter)	Novagen, USA
pET23a (+)	*E. coli* expression vector, (C-terminal His-tag, Amp^r^, T7 promoter)	Novagen, USA
pJET1.2/blunt	*Bla*, *rep(pMB1)*, *eco47IR*	Thermo Fisher Scientific, Germany
pJQ200mp18Tc	Tc^r^, *sac*B, suicide vector for gene deletion	Pötter et al. 2005
pET19b::*tctC*_44430	*E. coli* expression vector (N-terminal His-tag, Amp^r^, T7 promoter) expressing *tctC* (VPARA_44430)	This study
pET19b::*tctC*_01760	*E. coli* expression vector (C-terminal His-tag, Amp^r^, T7 promoter) expressing *tctC* (VPARA_44430)	This study

### Protein expression

In silico analysis of the TBEA6 genome revealed that the bacterium possesses 130 copies of *tctC* genes which encode for proteins homologues of TctC proteins used for the transport of different substrates. In silico analysis was done using the NCBI-Basic Local Alignment Search Tool (BLAST), and molecular biology tools such as multiple sequence alignment, Seqbuilder pro present in the DNA star software. BLAST-P search of TctC unravelled eight TctC proteins with more than 40% similarity to AdpC proteins (Table 2). Rosa et al. (2017) reported that AdpC proteins are orphan periplasmic-binding proteins from the TTT family. AdpC proteins are present in *Rhodopseudomonas palustris* and are involved in the transport of the substrate pimelate. Fortunately, pimelate exhibits a high structural similarity with TDP; hence we selected eight *tctC* genes of strain TBEA6 for protein expression and further identification of protein-substrate/protein-ligand (TctC-TDP) interactions.

**Table 2:** List of TctC proteins identified in *Variovorax paradoxus* strain TBEA6 exhibiting more than 40% amino acids similarity with AdpC proteins

S. No	Locus tag	Gene ID	Gene size (bp)	Protein (kDa)	Similarity with AdpC
1	VPARA-41790	2599562266	1017	36.6	44%
2	VPARA-46980	2599562784	969	35.5	43%
3	VPARA-27030	2566560789	987	36.1	42%
4	VPARA-01760	2599558252	981	35.9	41%
5	VPARA-39650	2599562052	993	36.4	41%
6	VPARA-55150	2599563606	978	35.8	41%
7	VPARA-14030	2599559489	996	36.5	41%
8	VPARA-44430	2599562531	990	36.3	41%

AdpC proteins are involved in the transport of pimelate in *Rhodopseudomonas palustris.* Pimelate had high structural similarity with TDP

### DNA isolation and PCR amplification

Chromosomal DNA of strain TBEA6 was isolated using the NucleoSpin Tissue kit (Macherey-Nagel, Germany) according to the manufacturer’s protocol. Eight *tctC* genes located in various locus tags were amplified from total genomic DNA by polymerase chain reaction (PCR). The primers were designed with the SeqBuilder (DNASTAR) and costume synthesized by MWG-Biotech AG (Ebersberg, Germany). Only one forward primer, but two different reverse primers (one for pET19b vector and one for pET23a vector) were used for each gene amplification. Nucleotide sequences of all primers were provided in (Table S1). The primers were designed in a way that the fragment can later be cut by restriction enzymes and ligated into the multiple cloning site (MCS) of pET19b and pET23a vectors. PCR reactions were carried out with Phusion high-fidelity DNA polymerase (Thermo Fisher Scientific, USA) or with Biomix (Bioline, UK) using the Omnigene HBTR3CM DNA thermal cycler (Hybaid, Germany). A Gel Extraction Kit (PEQLAB Biotechnologie GmbH, Germany) was used to purify the PCR products from agarose gel. Purified products were used for cloning in various vectors.

### Cloning

Intermediate cloning was done using the PCR products and the vector pJET1.2/blunt (CloneJET Kit, Thermo Scientific) based on the supplier’s instructions. The blunt-end PCR products were ligated directly into the vector which is an easy and reliable method because the amplified genes safely ligate and replicate in the plasmid. Expression vectors pET19b and pET23a were used for the T7 promoter-based heterologous expression. The *tctC* genes were cut out from the hybrid plasmid pJET1.2::*tctC* with appropriate restriction endonucleases (*Nde*I, *Xho*I, *Bam*HI, and *Hind*III) and purified with the peqGOLD Gel Extraction kit. Each purified *tctC* gene was cloned into both, the pET19b and the pET23a vectors. Whereas pET19b contains the N-terminal His tag, pET23a contains the C-terminal His tag. Ligation into the pET vectors occurred due to complementary single-stranded sticky ends between vector and fragments. The ligation products pET19b::*tctC* or pET23a::*tctC* were then transformed into *E. coli* Top10 competent cells. Suitable transformants were selected by growing cells on LB agar plates containing ampicillin. Grown cells were subjected to colony PCR, and the PCR amplified products were loaded on agarose gels to verify the bands. Hybrid plasmids from suitable transformants were isolated using the PeqGOLD Plasmid Miniprep Kit I (Peqlab, Erlangen, Germany), analyzed by sequencing (MWG-Biotech AG, Germany), and used for transformation into *E. coli* BL21 competent cells (New England BioLabs, MA). For sequencing, samples were sent to Eurofins Genomics in a total volume of 17 μl. Obtained sequence results were monitored by Seqlab software (Göttingen, Germany) to check the gene sequence.

### Protein purification

Heterologous expression of genes in *E. coli* BL21 cells was accomplished by cultivation in an auto inductive (ZYP-5052) medium or in LB medium. To the LB medium, 0.4 mM IPTG was added to induce the cells. ZYP medium (100 ml) supplemented with ampicillin was inoculated with 1% (vol/vol) of preculture. Initially cells were cultivated at 37 °C for 4 h and then at 20 °C. The cultures were grown on a rotary shaker at 130 rpm for about 18 h. Cells were harvested by centrifugation (9000 ×*g*, 4 °C, 20 min), washed twice with sterile saline, and resuspended in the appropriate buffers. Cells were resuspended in 50 mM Tris-HCl buffer (both pH 7.4), containing 20 mM imidazole and subsequently interrupted by 4 times passage through a French press (100 × 10^6^ Pa). Centrifugation (9000 ×*g*, 4 °C, 60 min) was done to obtain the supernatants containing soluble protein fractions from crude extracts. The supernatant was used for TctC protein purification. All buffers were used as suggested by the His Spin Trap affinity columns (GE Healthcare, UK) providers for the purification of histidine-tagged fusion proteins. Tris-HCl buffer (pH 7.4) with 20 mM imidazole was used for column equilibration. Buffers with 50 mM Tris-HCl and various imidazole concentrations (40 and 100 mM) were used for the first and second washing steps to achieve better purity. Finally, elution was carried out using an elution buffer containing 500 mM imidazole. At the end protein was transferred to sodium phosphate buffer (pH 7.4) after removing imidazole in Vivaspin 500 columns (Sartorius AG, Germany). In most cases, bacteria showed higher protein expression with ZYP medium than by inducing with IPTG. Protein concentrations were determined as described by Bradford (1976) using a spectral photometer (Thermo Spectronic, USA) by measuring OD at 595 nm.

### SDS-PAGE

Protein samples were mixed with SDS loading buffer for SDS-PAGE analysis. Protein boiling with SDS along with a reducing agent (DTT or β-mercaptoethanol) denatured the protein. Gels were casted with 11.5% separating gel and 4% collecting gel. From each fraction (crude extract, lysate, flow through, washing fractions), 40 μg of protein samples were loaded onto the gel; from the eluted fractions samples, containing 5 μg protein were loaded on the gel. A color prestained protein standard (10 μl) with broad range from 11 to 245 kDa from NEB was also loaded. The gel was first run at 40 mA until the samples from the collecting gel were transferred to the separating gel. The gel was then allowed to run to completion at 80–100 mA. The finished SDS-PAGE gels were incubated for 5–7 min in coomassie blue staining solution and then decolorized overnight with 10% acetic acid. After decolorization, the gels were scanned with the Epson scanner and the images were captured.

### Thermoshift assays

The stabilizing effect of ligand (TDP) on TctC proteins was evaluated by thermoshift assays. Thermoshift assays were done using 1 μM of TctC protein, TDP or gluconate as ligand at various concentrations (from 0.1 to 3000 μM), and 5 × Sypro Orange (5000 × stock solution, Sigma-Aldrich, USA). The final volume of the samples containing all components was made to 20 μl using sodium phosphate buffer (20 μM, pH 7.4). Samples were added into 48-well plates and sealed with optical adhesive films (Applied Biosystems, USA). A StepOne real-time PCR system (Applied Biosystems, USA) was used to record the fluorescence according to the protocol from Vivoli et al. (2014). A temperature range from 20 to 90 °C was applied during the process. Controls were made without substrate or protein. Assays were done in triplicates. The Protein Thermal Shift Software 1.3 (Applied Biosystems, USA) was used to investigate the results.

### Deletion mutants

The suicide plasmid technique was used to obtain defined deletion mutants (Simon 1984). Deletion of the *tctC* genes in *V. paradoxus* (VPARA-44430 and VPARA-01760) was accomplished by cloning the upstream and downstream flanking regions of the particular gene into the *Xba*I restriction site of the suicide plasmid pJQ200mp18Tc. Upstream (484 bp) and downstream (950 bp) fragments of *tctC* genes were amplified by using the primers XbaI-up tctC/EcoRI-up tctC and EcoRI-down tctC/XbaI-down tctC, respectively. The subsequent fragments were restriction digested and ligated to produce a 1434-bp fragment. The constructs were joined into the suicide plasmid pJQ200mp18Tc, using pJET1.2/blunt as a subcloning vector. The us/ds flank and the pJQ vector were digested with the same restriction enzyme *Xba*I and then ligated. The resulting gene replacement plasmids (pJQ200mp18Tc::*ΔtctC-*44430 and pJQ200mp18Tc::*ΔtctC-*01760) were multiplied in *E. coli* TOP10 and then transferred to *E. coli* S17-1 for mobilization into the recipient *V. paradoxus* strain TBEA6 by the spot agar-mating technique (Friedrich et al. 1981). Deletion mutants were selected on nutrient broth plates comprising 10% sucrose and MSM agar plates containing gluconate as a carbon source. The antibiotic tetracycline (12 μg/ml) was added in MSM agar plates. Confirmation of a correct second homologous recombination event and ex situ integration of the target genes was done by PCR. For this, oligonucleotides that bound outside the flanking regions and the oligonucleotides that bound inside the target gene sequence were used. Moreover, amplified fragments covering the proximity of the deleted gene were verified by sequence analysis (Quandt and Hynes 1993; Pötter et al. 2005).

### Growth experiments

Strain TBEA6 was able to use gluconate or TDP as carbon and energy source on solid plates, so it was cultivated for 1 week in liquid cultures at 30 °C in MSM or nutrient broth. MSM was supplemented with 60 mM gluconate or 60 mM TDP as carbon source, respectively. Erlenmeyer flasks with baffles were used in liquid cultures to guarantee an optimal oxygen supply with the flasks incubated on a rotary shaker at 130 rpm (New Brunswick Scientific Co. Inc. USA). Both, the wild-type and the mutant strains were used for growth experiments. Controls without bacterial cells were also done. Optical density was measured at various time periods using a Klett Summerson photometer (Klett Units) to determine the growth. Growth experiments were performed in triplicates; the results were presented as average and standard deviation.

## Results

**Fig 1:**
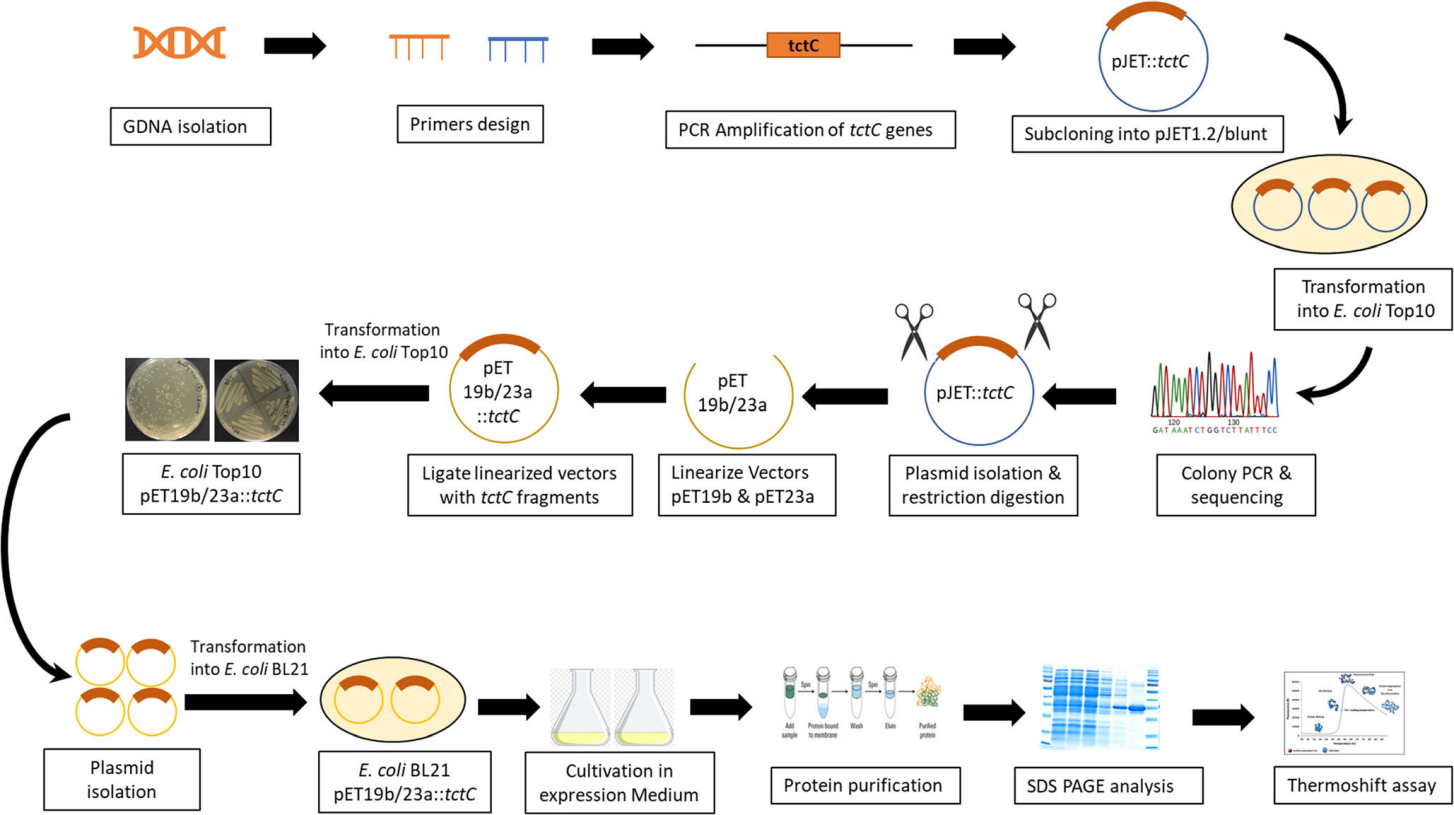
Overview of experimental methodology

Eight *tctC* genes were selected, amplified from genomic DNA of TBEA6, heterologously expressed in *E. coli* BL21 cells, and protein purifications were done. Subsequently, protein-ligand interactions were monitored using thermoshift assays. The experimental steps conducted in this study were summarized in Fig. 1.

### Heterologous expression and purification of TctC

**Fig 2:**
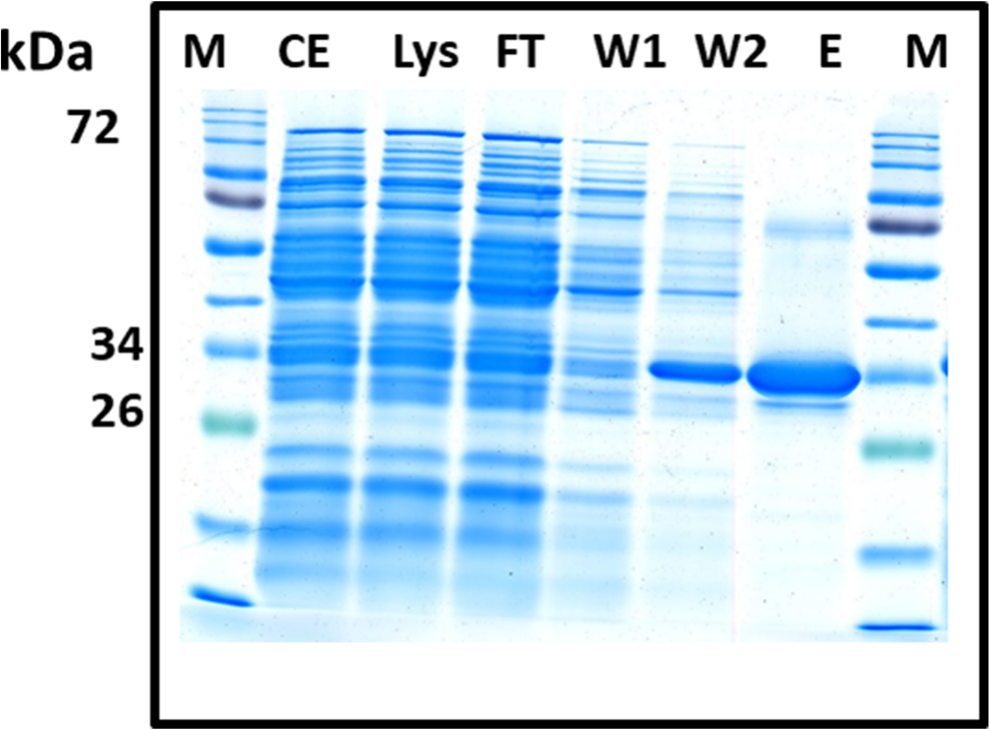
SDS-PAGE gel image of the purified TctC-44430 protein. The gene *tctC*-44430 was heterologously expressed in *E. coli* BL21 cells. Cells were grown in auto-induction medium, and protein purification was done through His Spin Trap affinity columns. Except elution, amounts of 40 μg protein were applied onto the gel from each sample. Elution fractions comprising 5 μg protein were applied onto the gel. M: marker; CE: crude extract; Lys: lysate; FT: flow through; W1: wash1; W2: wash2; E: elution

After genomic DNA isolation from the wild-type strain TBEA6, eight *tctC* genes, which exhibit high similarity with the AdpC protein, were selected for PCR amplification. During PCR, different annealing temperatures were used for amplification of the different genes. PCR results showed a single band with gene length of about 1000 bp in agarose gel electrophoresis. Before heterologous expression in *E. coli* BL21 cells, gene sequences were verified using Seqlab software, and it was confirmed that no mutations had occurred in the nucleotide sequence. Two types of expression vectors (pET19b and pET23a) were used, and almost similar protein concentrations were obtained with both vectors. Two types of media, i.e., ZYP-5052 and LB medium, were used for cultivation of cells and for protein expression. His Spin Trap affinity columns were used to purify the protein. Protein concentrations were determined using the Bradford method. *E. coli* BL21 cells, which harbor pET::*tctC*-44430 cultivated in ZYP medium, showed higher protein concentrations (crude extract: 1.81 mg/ml, lysate: 1.79 mg/ml, flow through: 1.25 mg/ml, wash1: 1.12 mg/ml, wash2: 0.85 mg/ml, and elution: 0.7 mg/ml). Similarly, cells with locus tag VPARA-01760 also showed significant protein concentration (crude extract: 1.74 mg/ml, lysate: 1.65 mg/ml, flow through: 1.31 mg/ml, wash1: 0.95 mg/ml, wash2: 0.89 mg/ml, and elution: 0.75 mg/ml). Different fractions obtained during protein purification were loaded on the SDS-PAGE gel to verify the purity and the molecular weight. The target protein (TctC) with locus tag VPARA-44430 had a molecular weight of 36 kDa. A SDS gel image of various fractions is shown in Fig. 2. Initially many bands were observed in elution buffer, but later washing conditions were optimized to get rid of non-specific bands.

### Thermoshift assays

**Fig 3:**
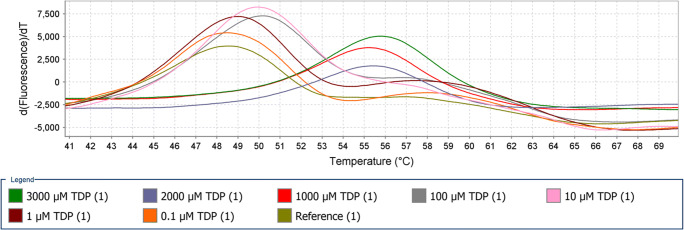
Thermal shift analysis of TctC-44430 protein along with the ligand TDP. Significant shift in the melting temperature of TctC was observed when it binds with TDP at various concentrations. The experiments were done in triplicates

**Fig 4:**
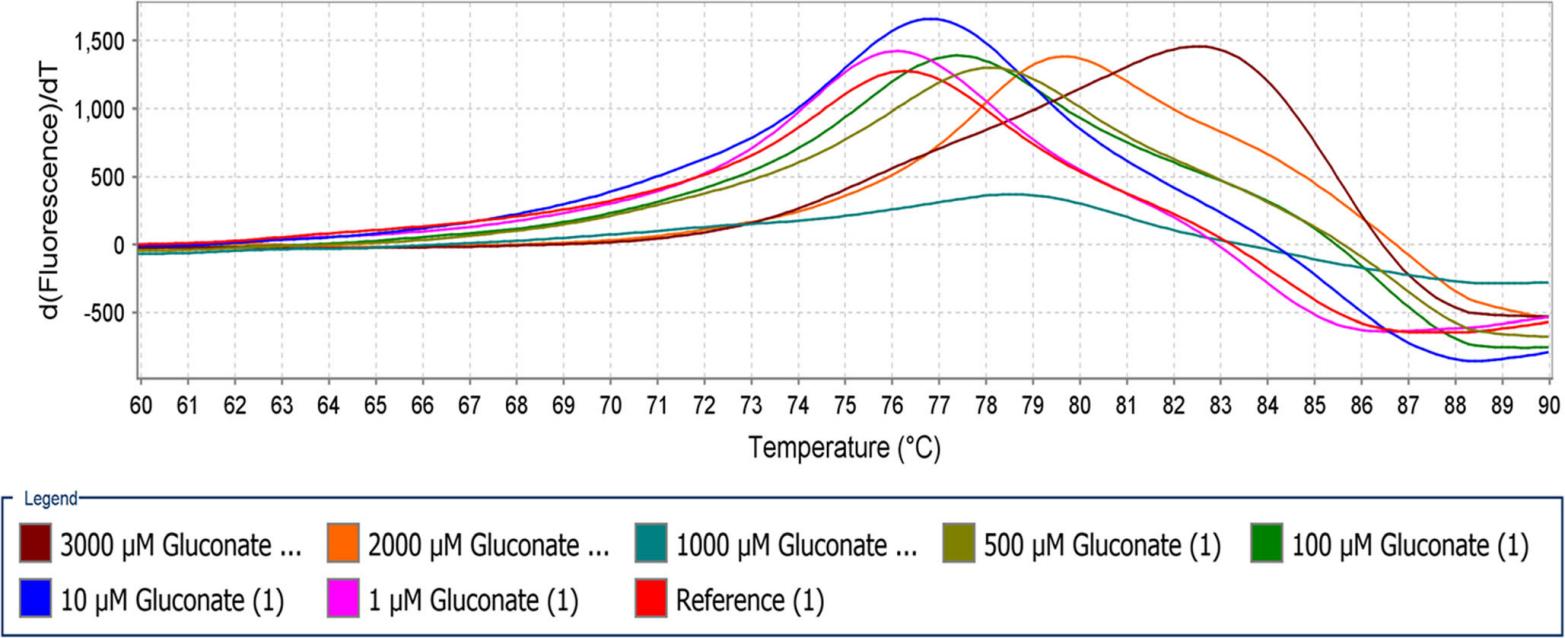
Thermal shift analysis of TctC-44430 protein along with the ligand gluconate. Significant shift in the melting temperature of TctC was observed when it binds with gluconate at various concentrations. The experiments were done in triplicates

Thermoshift assays were used to determine whether the TctC proteins are involved in the binding of TDP or not. If the TctC binds to TDP, its thermal stability will increase, and the melting temperature of TctC-TDP complex rises. The dye SYPRO orange binds to the hydrophobic side chains of TctC during the melting process. Two types of ligands (TDP or gluconate) at different concentrations (from 0.1 to 3000 μM) were applied for each protein. Out of the eight investigated proteins, a temperature shift was only observed with two proteins (locus tags VPARA-44430 and VPARA-01760). For the protein with locus tag VPARA-44430, the most substantial shifts were detected at TDP concentrations between 1000 and 3000 μM. The melt curve was observed at temperatures of 56 °C for 3000 μM concentration, and at 55.5 °C for 2000 and 1000 μM concentrations. This protein showed melt curves with TDP concentrations of 10 and 100 μM at same temperature, 50 °C. At lower concentrations of TDP, 1 and 0.1 μM melt curve was observed at temperatures of 49 C and 48.5 °C, respectively. At lower concentrations, lower but noticeable temperature shifts occurred in comparison to the control (Fig. 3). The same protein was used to conduct thermal shift analysis using gluconate as substrate at different concentrations. Very clear temperature shifts were observed at concentrations of 3000 μM at 83 °C followed by 2000 μM at 79.5 °C, 500 μM at 78.5 °C and 100 μM at 77.5 °C. Lower gluconate concentrations such as 1 and 10 μM showed temperature shifts at 77 and 76.5 °C, respectively (Fig. 4). The other protein with locus tag VPARA-01760 also showed temperature shifts with TDP or gluconate (Fig. S1 and S2). Although the shifts were not so clear like with the protein with locus tag VPARA-44430, clear shifts were observed when compared with the control. Even although TctC with locus tag VPARA-44430 showed interactions with TDP or gluconate in thermoshift assays, mutant was generated, and growth studies were performed to get more details about which substrate is binding to the TctC protein and transport in to *V. paradoxus* TBEA6.

### Growth experiments

**Fig 5:**
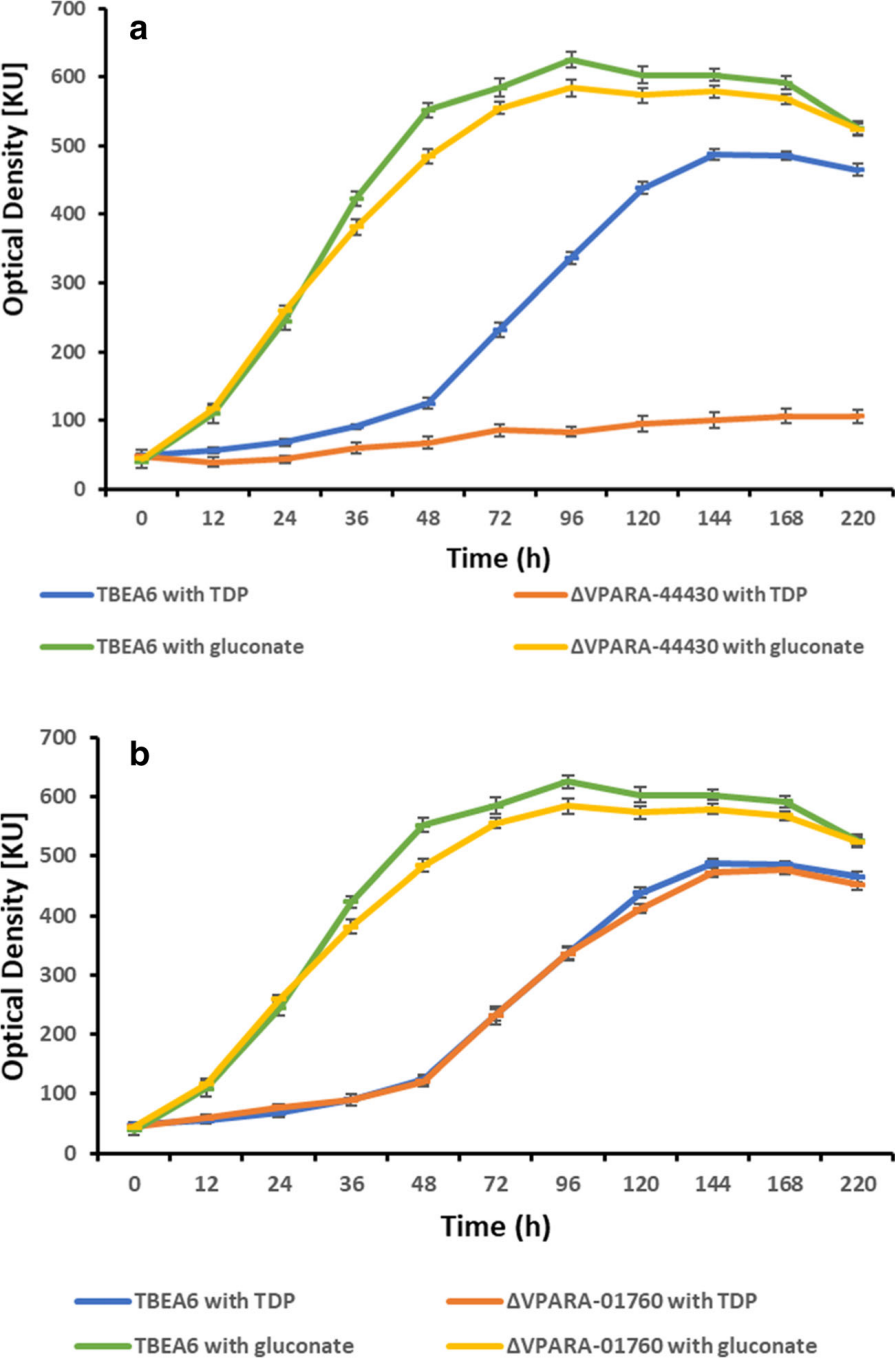
Growth curves of the wildtype (*Variovorax paradoxus* TBEA6); (A) mutant (∆VPARA-44430); and (B) mutant (∆VPARA-01760) strains using TDP or gluconate as sole carbon source. Both carbon sources were separately added in liquid MSM at a concentration of 60 mM. Triplicate experiments were done, and error bars are shown. KU indicates - Klett Units

Two TctC proteins (locus tags VPARA-44430 and VPARA-01760) out of eight proteins showed a significant shift in their melting temperatures when they interact with TDP. Therefore, the responsible genes were deleted in the genome of TBEA6, and single deletion mutants were subsequently generated. The reason to generate mutants was to find out whether the proteins encoded by these two *tctC* genes are really involved in the transport of TDP or not and also to compare the growth behavior of these two mutants with wild type using TDP as carbon source. Relevant deletion mutants were selected on nutrient broth agar plates containing 10% sucrose (showed growth) and on MSM agar plates containing gluconate and the antibiotic tetracycline (showed no growth). Confirmations of a correct second homologous recombination event and ex situ integration of the target genes were done by PCR analyses. Correct mutants showed no PCR product with internal primers and one PCR product with external primers. Internal primers bind inside the gene; external primers bind outside of the flanking regions. Moreover, amplified fragments covering the proximity of the deleted gene were verified by sequence analysis. Growth experiments were carried out after achieving deletion of the *tctC* genes (∆VPARA-44430 and ∆VPARA-01760). The wild-type strain TBEA6 was able to use gluconate or TDP as carbon and energy source on solid plates. One of the deletion mutants (∆VPARA-44430) was unable to utilize TDP, but utilized gluconate. The other mutant (∆VPARA-01760) was able to utilize both, TDP or gluconate, as sole carbon sources, like the wild type. The *tctC* gene existing in the locus tag VPARA-44430 was of special interest, as it seemed to be involved in TDP transport. Both the wild-type and mutant strains were separately cultivated for 1 week in liquid cultures at 30 °C in MSM by supplementing with 60 mM gluconate or 60 mM TDP as carbon sources. Erlenmeyer flasks with baffles were used in liquid cultures, and the flasks were incubated on a rotary shaker at 130 rpm. In liquid growth experiments, the deletion mutant ∆VPARA-44430 did not show any growth with TDP, whereas growth was observed with gluconate (Fig. 5a). When the cells were grown with TDP, the wild type entered the exponential phase after 48-h cultivation. A clear difference in the growth of the mutant and the wild-type strains was noticed from this point. After 48-h growth, the stationary phase starts with wild type at 144 h with OD of 487 KU, whereas the mutant did not show any increment in growth. Cells of the mutant were unable to reach the optical density of the wild type. Contrary to this, both, the wild-type and mutant strains cultivated with gluconate, showed an almost similar growth pattern. Wild type and mutant entered the exponential phase after 12 h with an OD of 110 KU. After that both strains showed rapid growth, with an OD value of 244 KU reached after 24 h. The OD value reached after 96-h cultivation was 625 KU; afterwards cells of both strains entered the stationary phase. In case of deletion mutants, lacking *tctC* with locus tag VPARA-01760 showed almost similar growth like wild-type strain using TDP or gluconate. No significant difference in growth was observed with wild-type and mutant strains (Fig. 5b).

## Discussion

Utilization of TDP by *V. paradoxus* TBEA6 was previously investigated and proved in our laboratory (Heine et al. 2019; Brandt et al. 2014; Schürmann et al. 2013; Bruland et al. 2009). TDP is first cleaved into 3-hydroxypropionate (3-HP) and 3-mercaptopropionate (3-MP), by an flavin adenine dinucleotide (FAD) linked oxidase (Fox) (Brandt et al. 2014; Bruland et al. 2009). The 3-MP is then oxygenated by a 3-MP dioxygenase yielding 3-sulfinopropionate (3-SP). The acyl-CoA-transferase (ActTBEA6) investigated by Schürmann et al. (2013) can catalyze the transformation of 3-SP to the corresponding CoA thioester, 3SP-CoA (Brandt et al. 2014; Schürmann et al. 2013). Subsequent removal of the sulfur moiety is catalyzed by a desulfinase, Acd, yielding sulfite, and propionyl-CoA. Propionyl-CoA enters in to central metabolism via three different possible pathways, i.e., the malonate semialdehyde pathway, the methylmalonyl-CoA pathway, and the methylcitrate cycle (Heine et al. 2019). In the first pathway, dehydrogenases and hydratases convert the propionyl-CoA in to acetyl-CoA. Methylmalonyl-CoA mutase catalyze the reactions and produce succinyl-CoA in the second pathway. In the third pathway, dehydratase enzymes involved and produce methylcitrate and methylisocitrate. Succinyl-CoA and acetyl-CoA from all three pathways enter into central metabolism. Recently, proteomic analysis conducted by Heine et al., (2019) provided new insights in TDP metabolism. Even though *tctC* genes from *V. paradoxus* TBEA6 were heterologously expressed in *E. coli*, it could not become a TDP utilizer, because *E. coli* does not produce the enzymes which can degrade TDP. An intracellular FAD-dependent oxidoreductase, which putatively converts TDP into 3-mercaptopropionate and 3-hydroxypropionic acid, was identified (Wübbeler et al. 2015). Previous study explored that import of TDP may occur by TTT systems (Wübbeler et al. 2015). In their study 32 mutants were unable to grow with TDP as sole carbon source. In most of these mutants Tn5::mob insertions were mapped within a gene encoding a protein related to the TTT family (Wübbeler et al. 2015). Based on this information, an in silico analysis was done in this study to identify genes which encode the proteins belonging to the TTT family. In silico analysis revealed that about 130 *tctC* gene homologues are present in the genome of *V. paradoxus* TBEA6. Among them eight *tctC* genes were selected which have high structural similarity with the AdpC protein in *Rhodopseudomonas palustris* (Table 2), heterologously expressed, and protein-ligand interactions were monitored using thermoshift assays.

Almost similar protein concentrations were obtained with both the vectors, i.e., pET19b and pET23a. *E. coli* BL21 cells, which harbor pET::*tctC*-44430 and cultivated in ZYP medium, showed higher protein concentrations. SDS-PAGE gel showed the purity and the molecular weight of each protein. Expression and analysis of TctC proteins are gaining importance because these proteins are putatively involved in the transport of many substrates through the TTT system. TTT system was first examined in *Salmonella typhimurium*. TctA is an integral membrane protein with 12 transmembrane domains comprising highly conserved motifs. TctB is also a membrane protein but possesses only four transmembrane domains, which are less conserved than TctA (Tamber et al. 2006). TctC is the periplasmic binding receptor protein, able to mediate the import by interactions with TctA and TctB (Huvent et al. 2006). The import is typically regulated by TctD and TctE. TctE is a sensor histidine kinase and interacts with TctC and the substrate which leads to the phosphorylation of TctD in the process of signaling cascade. TctD acts as a transcriptional regulator after the phosphorylation process (Antoine et al. 2005). The TctDE complex guarantees a transcription of the genes for the subunits of TctABC. In this system, fast variations will occur based on substrate concentration due to the continuous availability of TctC in the periplasm (Antoine et al. 2005). Homologs of *tctC* genes were explored in species of the genera *Bordetella*, *Ralstonia*, *Advenella*, and *Variovorax* (Pohlmann et al. 2007; Wübbeler et al. 2014).

Out of the eight investigated proteins, a temperature shift was observed with two proteins (locus tags VPARA-44430 and VPARA-01760) using the ligands TDP or gluconate. Reports are available about determinations of protein-ligand interactions in various bacteria. Schäfer et al. (2019) conducted thermoshift assays using protein TctC1 with locus tag MIM-c39190 in *A. mimigardefordensis* DPN7T and reported that TctC1 showed a substantial shift in the melting temperature with citrate, whereas no shift occurred with the ligand α-ketoglutarate. Heine et al. (2019) used TDP-CoA as ligand and the two proteins Ech-20 and Ech-30 from *V. paradoxus* TBEA6 in thermal shift assays to explain a protein ligand interaction. They reported that in presence of TDP-CoA a shift in the melting temperature of Ech-20 was obtained, but no shift was obtained with TDP. Contrary to this, shift in the melting temperature of Ech-30 either with TDP-CoA or with TDP was not recorded. Rosa et al. (2019) reported that MatBAC system belongs to the TTT family in the photosynthetic bacterium *Rhodopseudomonas palustris* and is involved in C4 dicarboxylic acids transport. Thermal shifts profiles were recorded using the periplasmic binding protein MatC with the substrates malate, succinate, and fumarate.

Growth experiments denoted that the wild-type strain TBEA6 was able to use gluconate or TDP as carbon and energy source. One of the deletion mutants (∆VPARA-44430) was unable to utilize TDP, but utilized gluconate. The other mutant (∆VPARA-01760) was able to utilize both, TDP or gluconate, as the sole carbon source, like the wild type. Reports are available about growth of *V. paradoxus* strain TBEA6 using various substrates. Heine et al. (2019) used the TDP, succinate, and gluconate each at 60 mM concentration as carbon source for growth of strain TBEA6. Strain TBEA6 grew also well with 30 mM TDP (Wübbeler et al. 2015) and 20 mM sodium gluconate along with 1 mM TDP as carbon sources (Brandt et al. 2014).

In conclusion, eight putative *tctC* genes were successfully amplified from the genomic DNA of TBEA6, and the corresponding proteins were purified. Protein analyses showed a significant protein concentration with all purified proteins. Out of eight proteins, two TctC proteins with locus tags VPARA-44430 and VPARA-01760 showed a significant shift in their melting temperatures when they interacted with TDP or gluconate. In the growth experiments, wild-type strains were successfully grown with TDP or gluconate. Among the two different mutant strains, one mutant (∆VPARA-44430) was unable to grow with TDP, whereas the other (∆VPARA-01760) was able to grow with TDP indicating that *tctC* gene with locus tag VPARA-44430 is involved in the uptake of TDP. Contrary to this, both mutant strains showed growth with gluconate like the wild type.

## Supplementary Information

ESM 1 (PDF 489 kb)

## Data Availability

The authors declare that data supporting the findings of this study are available within the article and its supplementary information files.

## References

[CR1] Antoine R, Dubuisson JF, Drobecq H, Willery E, Lesjean S, Locht C (2003). Overrepresentation of a gene family encoding extra cytoplasmic solute receptors in *Bordetella*. J Bacteriol.

[CR2] Antoine R, Huvent I, Chemalal K, Deray I, Raze D, Locht C, Dubuisson JF (2005). The periplasmic binding protein of a tripartite tricarboxylate transporter is involved in signal transduction. J Mol Biol.

[CR3] Bradford MM (1976). A rapid and sensitive method for the quantitation of microgram quantities of protein utilizing the principle of protein-dye binding. Anal Biochem.

[CR4] Brandt U, Hiessl S, Schuldes J, Thurmer A, Wübbeler JH, Daniel R, Steinbüchel A (2014). Genome-guided insights into the versatile metabolic capabilities of the mercaptosuccinate-utilizing beta-proteobacterium *Variovorax paradoxus* strain B4. Environ Microbiol.

[CR5] Bruland N, Wübbeler JH, Steinbüchel A (2009). 3-Mercaptopropionate dioxygenase, a cysteine dioxygenase homologue, catalyzes the initial step of 3-mercaptopropionate catabolism in the 3,3-thiodipropionic acid degrading bacterium *Variovorax paradoxus*. J Biol Chem.

[CR6] Friedrich B, Hogrefe C, Schlegel HG (1981). Naturally occurring genetic transfer of hydrogen-oxidizing ability between strains of *Alcaligenes eutrophus*. J Bacteriol.

[CR7] Heine V, Berning CM, Lück J, Mikowsky N, Voigt B, Riedel K, Steinbüchel A (2019). The catabolism of 3,3’-thiodipropionic acid in *Variovorax paradoxus* strain TBEA6: A proteomic analysis. PLoS One.

[CR8] Hosaka M, Kamimura N, Toribami S, Mori K, Kasai D, Fukuda M, Masai E (2013). Novel tripartite aromatic acid transporter essential for terephthalate uptake in *Comamonas* sp. strain E6. Appl Environ Microbiol.

[CR9] Huvent I, Belrhali H, Antoine R, Bompard C, Locht C, Dubuisson JF, Villeret V (2006). Crystal structure of *Bordetella pertussis* BugD solute receptor unveils the basis of ligand binding in a new family of periplasmic binding proteins. J Mol Biol.

[CR10] Kim DY, Lütke-Eversloh T, Elbanna K, Thakor N, Steinbüchel A (2005). Poly (3-mercaptopropionate): a non biodegradable biopolymer. Biomacromol.

[CR11] Lütke-Eversloh T, Steinbüchel A (2004). Microbial polythioester. Macromol Biosci.

[CR12] Pohlmann A, Fricke WF, Reinecke F, Kusian B, Liesegang H, Cramm R, Eitinger T, Ewering C, Pötter M, Schwartz E, Strittmatter A, Voß I, Gottschalk G, Steinbüchel A, Friedrich B, Bowien B (2007). Genome sequence of the bioplastic-producing Knall gas bacterium *Ralstonia eutropha* H16. Nat Biotechnol.

[CR13] Pötter M, Müller H, Steinbüchel A (2005). Influence of homologous phasins (PhaP) on PHA accumulation and regulation of their expression by the transcriptional repressor PhaR in *Ralstonia eutropha* H16. Microbiol (SGM).

[CR14] Quandt J, Hynes MF (1993). Versatile suicide vectors which allow direct selection for gene replacement in Gram-negative bacteria. Gene.

[CR15] Rodriguez IC, Stöveken N, Satola B, Wübbeler JH, Steinbüchel A (2011). Aerobic degradation of mercaptosuccinate by the Gram-negative bacterium *Variovorax paradoxus* Strain B4. J Bacteriol.

[CR16] Rosa LT, Dix SR, Rafferty JB, Kelly DJ (2017). Structural basis for high-affinity adipate binding to AdpC (RPA4515), an orphan periplasmic-binding protein from the tripartite tricarboxylate transporter (TTT) family in *Rhodopseudomonas palustris*. FEBS J.

[CR17] Rosa LT, Dix SR, Rafferty JB, Kelly DJ (2019). A new mechanism for high-affinity uptake of C4-dicarboxylates in bacteria revealed by the structure of *Rhodopseudomonas palustris* MatC (RPA3494), a periplasmic binding protein of the tripartite tricarboxylate transporter (TTT) family. J Mol Biol.

[CR18] Sambrook J, Fritsch EF, Maniatis T (1989). Molecular cloning: a laboratory manual.

[CR19] Satola B, Wübbeler JH, Steinbüchel A (2013). Metabolic characteristics of the species *Variovorax paradoxus*. Appl Microbiol Biotechnol.

[CR20] Schäfer L, Berning CM, Wübbeler JH, Steinbüchel A (2019). A tripartite tricarboxylate transporter (MIM_c39170–MIM_c39210) of *Advenella mimigardefordensis* DPN7T is involved in citrate uptake. Int Microbiol.

[CR21] Schlegel HG, Kaltwasser H, Gottschalk G (1961). A submersion method for culture of hydrogen-oxidizing bacteria: growth physiological studies. Arch Mikrobiol.

[CR22] Schürmann M, Hirsch B, Wübbeler JH, Stöveken N, Steinbüchel A (2013). Succinyl-CoA:3-Sulfinopropionate CoA-transferase from *Variovorax paradoxus* strain TBEA6, a novel member of the class III coenzyme A (CoA)-transferase family. J Bacteriol.

[CR23] Simon R (1984). High frequency mobilization of gram-negative bacterial replicons by the in vitro constructed Tn5-mob transposon. Mol Genet Genomics.

[CR24] Simon R, Priefer U, Pühler A (1983). A broad host range mobilization system for in vivo genetic engineering: transposon mutagenesis in Gram negative bacteria. Nat Biotechnol.

[CR25] Tamber S, Maier E, Benz R, Hancock REW (2006). Characterization of OpdH, a *Pseudomonas aeruginosa* porin involved in the uptake of tricarboxylates. J Bacteriol.

[CR26] Vivoli M, Novak HR, Littlechild JA, Harmer NJ (2014). Determination of protein-ligand interactions using differential scanning fluorimetry. J Vis Exp.

[CR27] Winnen B, Hvorup RN, Saier MH (2003). The tripartite tricarboxylate transporter (TTT) family. Res Microbiol.

[CR28] Wübbeler JH, Hiessl S, Schuldes J, Thürmer A, Daniel R, Steinbüchel A (2014). Unravelling the complete genome sequence of *Advenella mimigardefordensis* strain DPN7T and novel insights in the catabolism of the xenobiotic polythioester precursor 3,3´-dithiodipropionate. Microbiol (SGM).

[CR29] Wübbeler JH, Hiessl S, Meinert C, Poehlein A, Schuldes DR, Steinbüchel A (2015). The genome of *Variovorax paradoxus* strain TBEA6 provides new understandings for the catabolism of 3,3′-thiodipropionic acid and hence the production of polythioesters. J Biotechnol.

[CR30] Xia Y, Wübbeler JH, Qi Q, Steinbüchel A (2012). Employing a recombinant strain of *Advenella mimigardefordensis* for biotechnical production of homopolythioesters from 3,3´-dithiodipropionic acid. Appl Environ Microbiol.

