# Supplementary material for: 3,3′-Thiodipropionic acid (TDP), a possible precursor for the synthesis of polythioesters: identification of TDP transport proteins in Variovorax paradoxus TBEA6
Source: Appl Microbiol Biotechnol. 2021 Apr 26;105(9):3733–43. doi: 10.1007/s00253-021-11294-y (PMC8102459; doi:10.1007/s00253-021-11294-y)
Supplement: Supplementary file 1 — (PDF 489 kb) [file 253_2021_11294_MOESM1_ESM.pdf]

Journal name: Applied Microbiology and Biotechnology

3,3′-Thiodipropionic acid (TDP), a possible precursor for the synthesis of Polythioesters: identification of TDP transport proteins in *Variovorax paradoxus* TBEA6

M. Venkateswar Reddy[1], Alexander Steinbüchel*[1,2]

[1]Institut für Molekulare Mikrobiologie und Biotechnologie, Westfälische Wilhelms-Universität Münster, 48149 Münster, Germany

[2]Environmental Sciences Department, King Abdulaziz University, Jeddah, Saudi Arabia

*Author for correspondence: Alexander Steinbüchel.
Institut für Molekulare Mikrobiologie und Biotechnologie, Westfälische Wilhelms-Universität Münster, 48149 Münster, Germany
Tel: +49-251-8339821, Fax: +49-251-8338388. E-mail: steinbu@uni-muenster.de

Running title: TDP transport in *Variovorax paradoxus* TBEA6

Abstract

3,3′-Thiodipropionic acid (TDP) is an antioxidant, which can be used as precursor carbon source to synthesize polythioesters. The bacterium *Variovorax paradoxus* TBEA6 can use TDP as a single source of carbon and energy. In the present study, experiments were carried out to identify proteins involved in the transport of TDP into the cells of strain TBEA6. Hence, eight putative *tctC* genes, which encode for the TctC proteins, were amplified from genomic DNA of TBEA6 using polymerase chain reaction and heterologously expressed in *E. coli* BL21 cells. Cells were grown in auto-induction medium, and protein purification was done using His Spin Trap affinity columns. Purity and molecular weight of each protein were confirmed by SDS-PAGE analysis. Protein-ligand interactions were monitored in thermoshift assays using the real-time PCR system. Two TctC proteins (locus tags VPARA-44430 and VPARA-01760) out of eight proteins showed a significant shift in their melting temperatures when they interact with the ligand (TDP or gluconate). The responsible genes were deleted in the genome of TBEA6 using suicide plasmid pJQ200mp18Tc and single deletion mutants of the two candidate genes were subsequently generated. Finally, growth of the wild type strain (TBEA6) and the two mutant strains (ΔVPARA-44430 and ΔVPARA-01760) were monitored and compared using TDP or gluconate as carbon sources. Wild type strains were successfully grown with TDP or gluconate. From the two mutant strains, one (ΔVPARA-44430) was unable to grow with TDP indicating that the *tctC* gene with locus tag VPARA-44430 is involved in the uptake of TDP.

Keywords: 3,3′-Thiodipropionic acid (TDP), deletion mutant, polythioesters, protein purification, thermoshift assay, *Variovorax paradoxus*.

Table S1: Oligonucleotides used for the amplification of *tctC* genes located at various locus tags in genomic DNA of *Variovorax paradoxus* TBEA6.

**Locus tag**	**Primer**	**Sequence 5'-3'**	**Use**
VPARA-41790	Forward	CATATGACTTCTTCAAATTTCAAGACGAACC	To amplify *tctC* at locus tag VPARA-41790
	Reverse1	GGATCCTTAGTCCACTTTTGCGCCGGAGTC	
	Reverse2	AAGCTTGTCCACTTTTGCGCCGGAGTC	
VPARA-46980	Forward	CATATGAAACTCCTGCGCCATCTCG	To amplify *tctC* at locus tag VPARA-46980
	Reverse1	CTCGAGTCAGTCCACTTTCGCGCCG	
	Reverse2	CTCGAGGTCCACTTTCGCGCCG	
VPARA-27030	Forward	CATATGAAAAGAAGACAACTGCTGGCTTG	To amplify *tctC* at locus tag VPARA-27030
	Reverse1	CTCGAGTCAATCCAGACGGATGTTCCGC	
	Reverse2	CTCGAGATCCAGACGGATGTTCCGC	
VPARA-01760	Forward	CATATGAACAAGACGCTCTCCTCATTCC	To amplify *tctC* at locus tag VPARA-01760
	Reverse1	CTCGAGCTATTCGGCAGTCGCGCC	
	Reverse2	CTCGAGTTCGGCAGTCGCGCC	
VPARA-39650	Forward	CATATGGTGACCTCGTATTCCGCACCC	To amplify *tctC* at locus tag VPARA-39650
	Reverse1	CTCGAGTCACTCGGCCTTGGCGC	
	Reverse2	CTCGAGCTCGGCCTTGGCGC	
VPARA-55150	Forward	CATATGTTGAAGTTCATGCATTCGCTTGC	To amplify *tctC* at locus tag VPARA-55150
	Reverse1	CTCGAGTCAGTCGAGGCTGGCGCC	
	Reverse2	CTCGAGGTCGAGGCTGGCGCC	
VPARA-14030	Forward	CATATGCCCTTCATCCAACGCG	To amplify *tctC* at locus tag VPARA-14030
	Reverse1	CTCGAGTCAGTCCGCGGTCGCCC	
	Reverse2	CTCGAGGTCCGCGGTCGCCC	
VPARA-44430	Forward	CATATGAATGCAACAACCAAGCGGC	To amplify *tctC* at locus tag VPARA-44430
	Reverse1	GGATCCTCAGTCCAGCTCGATCTTGTTGGTC	
	Reverse2	AAGCTTGTCCAGCTCGATCTTGTTGGTC	

The primers were designed in a way that the fragment can later be cut by restriction enzymes and ligated into the multiple cloning site of pET19b and pET23a vectors. Restriction enzyme *Nde*I was used for all the forward primers. Restriction enzyme *Xho*I was used for all the reverse primers except VPARA-41790 and VPARA-44430. Restriction enzymes *BamH*I (Reverse1) and *Hind*III (Reverse2) were used for the VPARA-41790 and VPARA-44430.

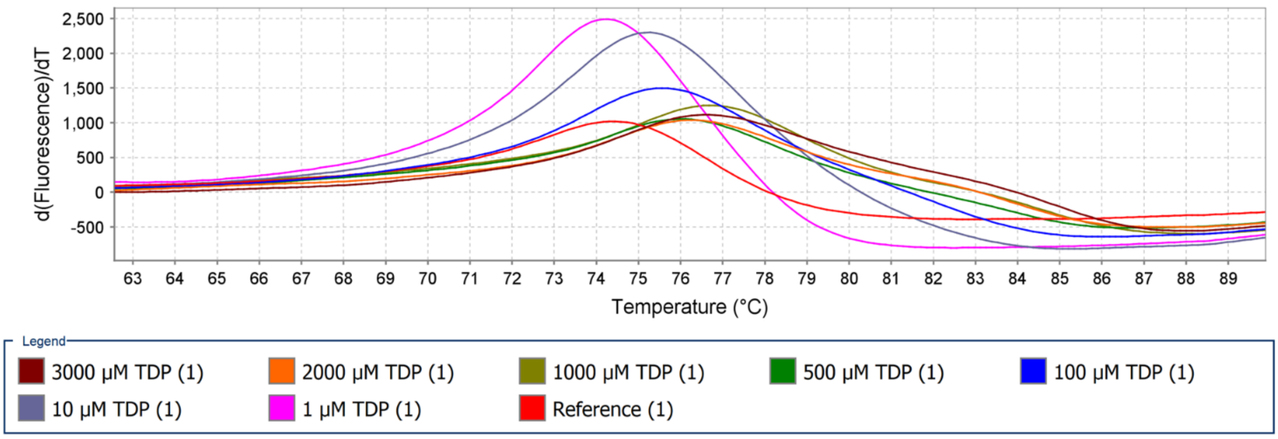

Fig S1. Thermal shift analysis of TctC-01760 protein along with the ligand TDP. A shift in the melting temperature of TctC was observed when it binds with TDP at various concentrations. The experiments were done in triplicates.

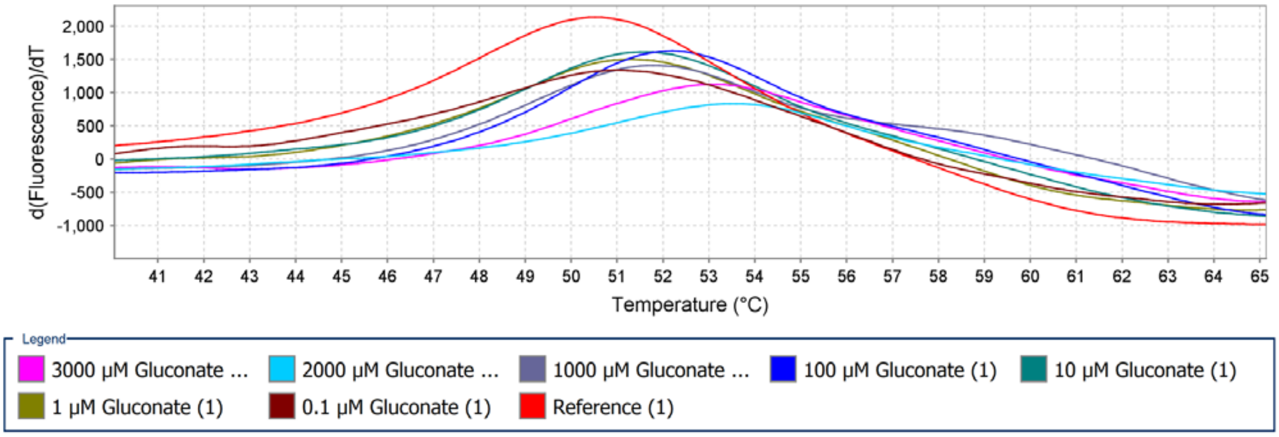

Fig S2. Thermal shift analysis of TctC-01760 protein along with the ligand gluconate. No significant shifts in the melting temperature of TctC were observed when it binds with gluconate at various concentrations. The experiments were done in triplicates.
